# Environmental Stability of SARS-CoV-2 on Different Types of Surfaces under Indoor and Seasonal Climate Conditions

**DOI:** 10.3390/pathogens10020227

**Published:** 2021-02-18

**Authors:** Taeyong Kwon, Natasha N. Gaudreault, Juergen A. Richt

**Affiliations:** Department of Diagnostic Medicine/Pathobiology, College of Veterinary Medicine, Kansas State University, Manhattan, KS 66506, USA; tykwon@vet.k-state.edu (T.K.); nng5757@vet.k-state.edu (N.N.G.)

**Keywords:** SARS-CoV-2, environmental stability, fomite, virus decay

## Abstract

Transmission of severe acute respiratory coronavirus 2 (SARS-CoV-2) mainly occurs through direct contact with an infected person via droplets. A potential role of contaminated surfaces in SARS-CoV-2 transmission has been suggested since the virus has been extensively detected on environmental surfaces. These findings have driven the investigation of virus stability on surfaces under several conditions. However, it remains unclear how long the infectious virus survives on surfaces under different climate conditions, which could play a role in predicting the seasonality of SARS-CoV-2. Therefore, the aim of this study was to estimate the virus stability and its biological half-life on various types of surfaces under indoor and seasonal climate conditions. This study revealed that SARS-CoV-2 survived the longest on surfaces under winter conditions, with a survival post-contamination on most surfaces up to 21 days, followed by spring/fall conditions, with a survival up to 7 days. Infectious virus was isolated up to 4 days post-contamination under indoor conditions, whereas no infectious virus was found at 3 days post-contamination under summer conditions. Our study demonstrates the remarkable persistence of SARS-CoV-2 on many different common surfaces, especially under winter conditions, and raises awareness to the potential risk of contaminated surfaces to spread the virus.

## 1. Introduction

Severe acute respiratory coronavirus 2 (SARS-CoV-2), which first emerged in December 2019 in a wet market in Wuhan, China, is responsible for the current pandemic. Although transmission of SARS-CoV-2 mainly occurs through infectious droplets or close contact with an infected person, virus droplets can survive and remain infectious on inanimate surfaces, which can contribute to the spread of the virus [1]. Previous studies showed that the virus remained infectious from hours to days on various types of surfaces under various temperature-controlled environmental conditions [2,3,4,5]. However, virus stability on surfaces under different climate conditions, which could be used to predict the seasonality of SARS-CoV-2, is poorly understood. In this study, we evaluated the stability of SARS-CoV-2 on different types of surfaces under indoor, summer, spring/fall, and winter conditions to estimate the biological half-life of the virus.

## 2. Results

**Figure 1:**
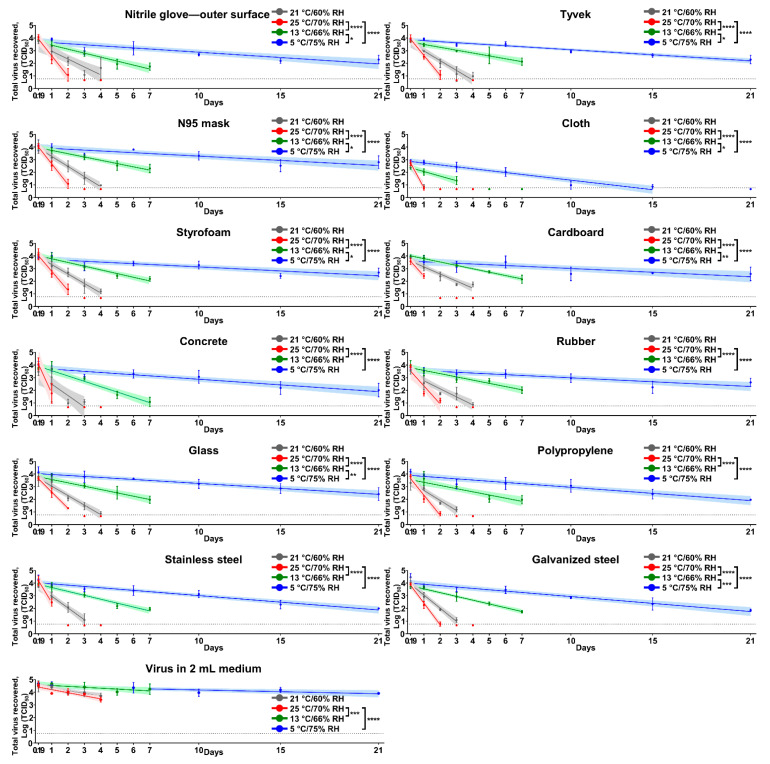
Stability of severe acute respiratory coronavirus 2 (SARS-CoV-2) on different types of surfaces. Each graph represents the virus decay on a defined surface. For this study, 50 μL of virus inoculum (5 × 10^4^ 50% tissue culture infectious dose (TCID_50_)) was added onto each material and dried for 4.5 h inside a biosafety cabinet. The virus survival was evaluated under the four following conditions: at 21 °C/60% relative humidity (RH) (grey), 25 °C/70% RH (red), 13 °C/66% RH (green), and 5 °C/75% RH (blue). The infectious virus was recovered at 0.19 (after drying period, and equal to 4.5 h), 1, 2, 3, and 4 days post-contamination (dpc) at 21 °C/60% RH and 25 °C/70% RH, 0.19, 1, 3, 5, and 7 dpc at 13 °C/66% RH and 0.19, 1, 3, 6, 10, 15, and 21 dpc at 5 °C/75% RH. Virus titer at each time point was expressed as mean log10 transformed titer with standard deviation. Virus titers at time points when at least one replicate was positive were incorporated to estimate linear regression models; the solid line and its shaded area represent an estimated best fit model and 95% confidence intervals, respectively. Limit of detection (LOD) in each titration assay was 10^0.968^ TCID_50_, and a negative result is represented as a half value of LOD, 10^0.667^ TCID_50_. The dash line shows LOD in triplicate, 10^0.767^ TCID_50_, when there was LOD in one replicate, but negative in two other replicates. Adjusted *p*-value between two slopes of linear regression models is represented as * (*p* < 0.05), ** (*p* < 0.01), *** (*p* < 0.001), and **** (*p* < 0.0001).

SARS-CoV-2 was relatively stable in medium throughout the study phase, showing a 1.17-log reduction of virus titer at 4 days post-contamination (dpc) at 25 °C/70% relative humidity (RH) (Figure 1). Surfaces were contaminated with 5 × 10^4^ 50% tissue culture infectious dose (TCID_50_) of SARS-CoV-2. After 4.5 h of incubation, virus titers on surfaces ranged from 10^2.4^ to 10^2.7^ TCID_50_ on cloth and 10^3.3^ to 10^4.2^ TCID_50_ on other materials; these virus titers served as the starting titers for the linear regression model. Under indoor conditions, infectious virus was recovered from cloth up to 1 dpc; from concrete, polypropylene, stainless steel, and galvanized steel up to 3 dpc; and from nitrile gloves, Tyvek, N95 mask, Styrofoam, cardboard, rubber, and glass up to 4 dpc. In contrast, viable virus disappeared quickly under summer conditions and was undetectable on cloth, cardboard, concrete, and stainless steel at 2 dpc, and on nitrile gloves, Tyvek, N95 mask, Styrofoam, rubber, glass, polypropylene, and galvanized steel at 3 dpc. However, the virus titer only slowly decayed over time under spring/fall conditions. Virus titers on surfaces ranged from 10^1.1^ to 10^2.3^ TCID_50_ at 7 dpc, except for cloth, with virus only detectable up to 3 dpc. Under winter conditions, we observed the longest survival time. The virus was able to survive up to 15 dpc on cloth and 21 dpc on most of the other surfaces. The half-life of SARS-CoV-2 on surfaces ranged from 3.5 to 12.86 h, 2.54 to 5.58 h, 17.11 to 31.82 h, and 47.94 to 121.78 h under indoor, summer, spring/fall, and winter conditions, respectively (Table 1). The virus survived significantly longer on all surfaces under spring/fall and winter conditions than summer conditions. Similarly, we found significant differences between winter and spring/fall conditions on nitrile gloves, Tyvek, N95 mask, cloth, Styrofoam, cardboard, glass, and galvanized steel.

## 3. Discussion

Potential modes of transmission of SARS-CoV-2 include direct contact with an infected person via droplets, inhalation of aerosol, or exposure to infectious body fluids or contaminated surfaces (fomite). To date, there has been no scientific report that has demonstrated SARS-CoV-2 infection via contaminated surfaces. However, the role of fomites in transmission of SARS-CoV-2 is debated because the virus has been detected on environmental surfaces as well as personal protective equipment in hospitals and households [6,7]. In addition, indirect transmission of SARS-CoV-2 has been supported by a cluster of SARS-CoV-2 infection cases in a shopping mall, in which contact tracing failed to find any evidence for direct contact to an infected person, only to the fact that they all shared the same facility [8]. In this respect, our study highlights the possible role of contaminated surfaces in SARS-CoV-2 transmissions because SARS-CoV-2 remained viable and infectious on surfaces for 1 to 4 days under indoor conditions (21 °C/60% RH), 1 to 2 days under summer conditions (25 °C/70% RH), over 7 days under spring/fall conditions (13 °C/66% RH), and up to 21 days under winter conditions (5 °C/75% RH). 

Van Doremalen et al. contaminated cardboard, plastics, and stainless steel with 50 μL of 10^5^ TCID_50_/mL virus inoculum (isolate USA-WA1/2020) and described the SARS-CoV-2 half-life, which ranges from 3.46 to 6.81 h at 22 °C/40% RH [3]. The study by Chin et al. calculated a biological half-life of 4.8 to 23.9 h at 22 °C/65% RH by adding 5 μL of virus culture (10^7.8^ TCID_50_/mL) to glass, banknotes, inner and outer mask layers, polypropylene, and stainless steel [2]. In another study, Biryukov et al. [5] investigated the effect of relative humidity, temperature, and droplet size on virus (isolate USA-WA1/2020; titer not provided) stability in saliva dried onto nonporous surfaces. SARS-CoV-2 decayed more rapidly when either humidity or temperature was increased, but droplet volume (1 to 50 μL) and surface type (stainless steel, plastic, or nitrile glove) did not impact the decay rate. The virus half-life ranged from 6.3 to 18.6 h at room temperature (24 °C) but was reduced to 1.0 to 8.9 h when the temperature was increased to 35 °C. We found the biological half-life on most surfaces at 21 °C/60% RH to be 6.93–12.86 h, but the virus is quickly inactivated on cloth, with a short 3.5 h half-life. The difference to other publications might be explained by the composition of the virus inoculum (e.g., fetal bovine serum concentration), the volume of inoculum, different preparation of the materials, and the different environmental conditions. However, our results, along with others, showed that SARS-CoV-2 is able to survive on most surfaces for several days under indoor conditions, which might play a potential role in virus transmission. The longest biological half-life of the virus was found in winter conditions, followed by spring/fall conditions and summer conditions; this suggests that virus stability on surfaces is highly dependent on seasonality. Prolonged virus survival in spring/fall and winter may have contributed to the drastic re-surge of daily COVID-19 cases in the US and in the Northern Hemisphere that started in the late fall of 2020 and could potentially contribute to new outbreaks and/or seasonal occurrence in the post-pandemic era, a scenario described for influenza virus and other human coronaviruses [9].

Our study showed a remarkable persistence of infectious SARS-CoV-2 on various types of surfaces, especially under winter climate conditions. However, virus stability was highly dependent on the substrate as well as temperature and humidity. Previous studies showed reduced virus stability in human nasal mucus and sputum when compared to culture medium [10] even at 4 °C/40% RH, whereas the addition of bovine serum albumin into the virus inoculum increased SARS-CoV-2 survival times [11]. In addition, exposure to simulated sunlight accelerated the inactivation of the virus on stainless steel [12], indicating that additional factors can play a role in SARS-CoV-2 survival on surfaces. The presence of infectious SARS-CoV-2 does not guarantee efficient transmission via fomites. Fomite transmission seems to depend on the quantity of surface contamination, virus survival on surfaces, mechanical transfer from surface to mucosa of naïve individuals, and exposure to the amount of virus over time. In-depth analyses that consider these other factors and not only virus stability are critical to understand the true risk of fomite transmission of SARS-CoV-2.

There are certain limitations to this study. First, we used the USA-WA1/2020 isolate, which was isolated in January 2020 from the first COVID-19 patient in the US. Since its first emergence at the end of 2019 and the subsequent global spread, SARS-CoV-2 has continuously evolved and accumulated mutations, which might have an impact on the environmental stability. Furthermore, the experimental design with a pre-determined time of termination (max. 21 days) resulted in samples (especially for winter conditions) which were harvested before the disappearance of infectious virus. Nonetheless, we were still able to successfully calculate the biological half-life of SARS-CoV-2 on surfaces and determine the influence of seasonality on virus decay. Lastly, the SARS-CoV-2 inoculum in this study was prepared in cell culture medium used to grow the susceptible cell line (VeroE6 cells); however, under natural conditions, the infectious virus is excreted in biological fluids (e.g., nasal secretions, saliva) from infected humans and animals. Therefore, the virus stability might be different in various types of biological fluids when compared to cell culture medium, which was used in this study.

In conclusion, our study determines the biological half-life of SARS-CoV-2 on diverse surfaces under different climatic conditions, which correlates to the potential risk of contaminated surfaces to spread the virus. It clearly demonstrates that the virus is more resistant under winter and spring/fall but not summer conditions. Therefore, the practice of good personal hygiene and regular disinfection of potentially contaminated surfaces remains a critical tool to minimize the risk of infection through contaminated surfaces.

## 4. Materials and Methods

SARS-CoV-2 stability was tested on 12 surfaces including nitrile glove (Kimber-ly-Clark Professional™ Kimtech™ G3 Sterile Sterling™ Nitrile Gloves, catalog number: 11822, Kimberly-Clark, Irving, TX, USA, purchased from Fisher Scientific, Hampton, NH, USA), Tyvek (DuPont™ Tyvek IsoClean Sleeves, Clean Processed & Sterile, White, catalog number: IC501BWHCS, DuPont, Wilmington, DE, USA, purchased from Thomas Scien-tific Swedesboro, NJ, USA), N95 mask (3M N95 mask 1870, Saint Paul, MN, USA), cloth (65% polyester and 35% cotton from local source, Manhattan, KS, USA), Styrofoam (50 mL centrifuge tube-foam rack, catalog number: 229422, CELLTREAT Scientific Products, Pep-perell, MA, USA), cardboard (inner packing, TPP T75 flask, catalog number: 90076, TPP, Trasadingen, Switzerland), concrete (Fast-setting concrete mix, catalog number: 100450, Quikrete, Atlanta, GA, USA, purchased from The Home Depot, Atlanta, GA, USA), rubber (Rubber-Cal, catalog number: 20-119-0062-36-012, Fountain Valley, CA, USA, purchased from The Home Depot), glass (Electron Microscopy Sciences, catalog number: 72227-01, Hatfield, PA, USA), polypropylene (biohazard autoclave bag, catalog number: 01-826-5, Thermo Fisher Scientific, Waltham, MA, USA), stainless steel (1/2 inch in diameter and 16 ga thickness, Metal Remnant Inc., Salt Lake City, UT, USA), and galvanized steel (M-D Building Products, catalog number: 56020, Oklahoma City, OK, USA, purchased from The Home Depot). Materials were cut into small pieces, washed, dried, and autoclaved (de-pending on material). To make concrete, the coarse aggregate was removed by a strainer, and the fine aggregate was mixed with water according to the manufacturer’s instruction. The mixture was then poured into a silicone mold and air-dried in a biosafety cabinet overnight. 

Each material surface was placed in a 6-well or 12-well plate, and 50 μL of virus inoculum consisting of 5 × 10^4^ TCID_50_ SARS-CoV-2 (strain USA-WA1/2020) in Dulbecco’s Modified Eagle’s Medium (DMEM) with 5% FBS was added onto each material. The positive control had the same amount of virus in 2 mL medium in a sealed 2 mL tube. The virus was air-dried inside a biosafety cabinet for 4.5 h at room temperature and room relative humidity (RH). The plate with the virus-contaminated material was then incubated under four different conditions in an environmental chamber (Temperature Test Chamber, Nor-Lake Scientific, Hudson, WI, USA): 21 °C/60% RH, 25 °C/70% RH, 13 °C/66% RH, and 5 °C/75% RH, environmental conditions simulating indoor setting, summer, spring/fall, and winter conditions for the Midwestern US, respectively. To calculate the average temperature and RH for each season, maximum and minimum temperature and relative humidity (RH) data at Manhattan, Kansas, was acquired from the National Service Forecast Office (https://w2.weather.gov/climate/index.php?wfo=top accessed on 11 May 2020) (Table 2). Climate conditions for spring and fall were combined since their average temperature and RH were similar. The assay for winter conditions was performed under 5 °C/75% RH instead of the real Midwestern average values (1 °C/67% RH) since this setting could be maintained in the chamber. The variations of temperature and RH were controlled at ±0.5 °C and ±2%, respectively. At each time point indicated, infectious virus was recovered in 2 mL media through vigorous vortexing for 10 s. Cardboard was soaked with media for 5 min and vortexed for 10 s. The recovered virus was titrated on Vero E6 cells, and virus titer was calculated by the Reed–Muench method. The assay was performed in triplicate. A best-fitting line was estimated using a linear regression model in order to calculate the half-life on each surface as a −log_10_(2)/slope in GraphPad Prism 9, San Diego, CA, USA. To determine the seasonal pattern of stability, analysis of variation (ANOVA) and subsequent Tukey’s multiple pairwise comparisons were performed to compare the slopes of linear regression under summer, spring/fall, and winter conditions.

## Figures and Tables

**Table 1:** Half-life of severe acute respiratory coronavirus 2 (SARS-CoV-2) on different types of surfaces under indoor and three seasonal conditions.

	21 °C/60% RH (Indoor Condition)			25 °C/70% RH (Summer Condition)			13 °C/66% RH (Spring/Fall Condition)			5 °C/75% RH (Winter Condition)		
	Half-Life (Hours)	95% CI (Hours)	*r^2^*	Half-Life (Hours)	95% CI (Hours)	*r^2^*	Half-Life (Hours)	95% CI (Hours)	*r^2^*	Half-Life (Hours)	95% CI (Hours)	*r^2^*
Nitrile gloves—outer surface	11.56	8.27, 19.21	0.69	4.42	3.5, 6.03	0.92	22.94	18.73, 29.63	0.88	85.71	65.37, 124.5	0.7
Tyvek	9.36	7.76, 11.79	0.89	4.57	3.84, 5.63	0.96	31.82	24.65, 44.82	0.81	90.59	78.19, 107.66	0.9
N95 mask	9.01	7.57, 11.12	0.91	4.4	3.64, 5.57	0.95	27.77	22.5, 36.27	0.87	106.37	76.68, 173.6	0.61
Cloth	3.5	2.77, 4.75	0.97	2.99	2.45, 3.84	0.98	19.94	13.94, 34.95	0.81	47.94	40.04, 59.74	0.88
Styrofoam	9.62	8.04, 11.98	0.9	4.75	3.73, 6.53	0.92	24.67	20.6, 30.73	0.9	112.91	82.75, 177.7	0.63
Cardboard	12.86	10.52, 16.54	0.88	5.03	3.5, 8,95	0.91	26.93	23.55, 31.42	0.95	121.78	81.65, 239.67	0.49
Concrete	7.96	5.25, 16.44	0.65	2.54	1.55, 6.98	0.83	17.11	14.38, 21.14	0.91	80.99	62.53, 114.9	0.73
Rubber	11.33	8.95, 15.45	0.83	5.03	3.63, 8.18	0.84	28.27	22.4, 38.32	0.84	115.74	84.04, 185.82	0.62
Glass	9.6	8.05, 11.89	0.91	5.58	4.72, 6.82	0.96	27.34	21.72, 36.87	0.84	92.03	72.82, 125.06	0.77
Polypropylene	9.02	7.22, 12.03	0.89	4.51	3.74, 5.68	0.95	28.75	21.52, 43.36	0.76	75.54	60.58, 100.31	0.79
Stainless steel	7.75	6.39, 9.86	0.92	3.41	2.36, 6.16	0.91	23.46	20.16, 28.08	0.93	70.06	59.43, 85.28	0.88
Galvanized steel	6.93	5.88, 8.43	0.94	4.19	3.68, 4.85	0.98	24.22	21.3, 28.08	0.95	67.21	55.49, 85.23	0.84
Positive control	35.54	23.19, 75.88	0.56	29.48	20.85, 50.39	0.68	100.68	52.35, 1346.89	0.3	263.37	155.41, 863.29	0.32

**Table 2:** Seasonal maximum and minimum temperature and relative humidity data for Manhattan, Kansas.

Season	Spring	Summer			Fall			Winter			Spring	
Month Year	May 2019	June 2019	July 2019	August 2019	September 2019	October 2019	November 2019	December 2019	January 2020	February 2020	March 2020	April 2020
Maximum temperature (°F)	73.6	86.9	91.8	86.7	88.1	63.7	54.5	48.6	42.8	47.5	60.1	67.9
Minimum temperature (°F)	52.9	61.9	68.3	68	65.9	39	27.2	23	22.3	21.8	37	39.8
Relative humidity (%)	73	67	66	76	69	66	60	68	73	61	67	59

## Data Availability

The data presented in this study are openly available in bioRxiv at https://doi.org/10.1101/2020.08.30.274241.
